# Short-term administration of growth hormone (GH) lowers blood pressure by activating eNOS/nitric oxide (NO)-pathway in male hypophysectomized (Hx) rats

**DOI:** 10.1186/1472-6793-5-17

**Published:** 2005-11-07

**Authors:** Henrik C Nyström, Natalia Klintland, Kenneth Caidahl, Göran Bergström, Anna Wickman

**Affiliations:** 1 Department of Physiology, Institute of Physiology & Pharmacology, The Sahlgrenska Academy, Göteborg University, P.O. Box 432, SE-405 30 Göteborg, Sweden; 2 Department of Clinical Physiology, Sahlgrenska University Hospital/SU, SE-413 45 Göteborg, Sweden

## Abstract

**Background:**

The aim of the study was to evaluate the acute and continuous (up to 14 days of treatment) effect of growth hormone (GH) on blood pressure (BP) regulation and to investigate the interplay between GH, nitric oxide (NO) and BP.

In un-supplemented and GH supplemented hypophysectomized (Hx) male rats as well as intact rats, continuous resting mean arterial blood pressure (MAP) was measured using telemetry. Baroreceptor activity and the influences of NO on BP control were assessed during telemetric measurement. Furthermore, basal plasma and urine nitrate levels and aortic endothelial nitric oxide synthase (eNOS) expression were analysed. Endothelial function as well as vascular structure in the hindquarter vascular bed was estimated using an *in vivo *constant-flow preparation.

**Results:**

Hypophysectomy was associated with decreased MAP (Hx: 83 ± 3 *vs *Intact: 98 ± 6 mmHg, p < 0.05) and heart rate (HR) (Hx: 291 ± 4 *vs *Intact: 351 ± 7 beat/min, p < 0.05). Endothelial dysfunction and reduced vasculature mass in the hindquarter vascular bed was found in Hx rats. GH substitution caused a further transient decrease in MAP and a transient increase in HR (14% and 16% respectively, p < 0.05). The reduction in MAP appeared to be NO dependent. Aortic eNOS expression was unchanged. GH substitution resulted in an impaired baroreceptor function. Two weeks of GH treatment did not normalise the BP, vascular structure and the endothelial function in the resistance vessels.

**Conclusion:**

GH substitution seems to have a short lasting effect on lowering blood pressure via activation of the NO-system. An interaction between GH, NO-system and BP regulation can be demonstrated.

## Background

Adult hypopituitarism and untreated growth hormone (GH) deficiency (GHD) is associated with endothelial dysfunction [1], decreased systemic formation of nitric oxide (NO) [2] and increased risk for cardiovascular morbidity and mortality [3] as compared to the normal population [4]. There are disparate reports on the effect on blood pressure (BP) in this condition; different studies have found normotension, hypotension or hypertension in GHD-patients (for review see [5]). Growth hormone replacement has been shown to reverse many of the adverse cardiovascular risk factors associated with GHD, including decreased systolic and diastolic BP, decreased vascular resistance [6], increased cardiac output (CO), increased heart rate (HR) [2,6], improved endothelial function [1,7] and increased NO formation [2].

Hypophysectomized (Hx) rats express decreased systolic BP and mean arterial blood pressure (MAP), CO and HR compared with intact rats [8,9]. As in human adult GHD, these rats show endothelial dysfunction and impaired vascular reactivity [10,11], while aortic endothelial nitric oxide synthase (eNOS) expression is not affected [12]. Furthermore, these rats also display decreased heart weights [12,13], and reduced vasculature mass in the muscle vascular bed suggesting an hypotropic remodelling of the cardiovascular system [13]. One week of GH substitution in Hx rats does not result in a change in systolic BP measured by the tail-cuff technique, whereas HR, heart weight and aortic eNOS expression are increased [12]. The resistance vessels (mesenteric artery) still demonstrate endothelial dysfunction [10]. Previous studies on BP regulation in Hx rats [10,12,13] have used less precise techniques to measure BP *i.e*. tail-cuff technique and therefore showed imprecise GH effect on acute and continuous BP regulation.

In the present study we aimed to investigate the acute and continuous (up to 14 days of treatment) effect of growth hormone substitution on blood pressure regulation. In order to measure the true, unstressed BP, we used a telemetry technique with implantable transmitters. We were also interested in investigating the interplay between GH, NO and BP. In order to do this we used a specific NOS blocker and measured the aortic eNOS expression, basal plasma and urine nitrate levels. In addition, baroreceptor activity was examined both early and late after onset of GH treatment. At the end of the study, endothelial function and the vascular structural properties in the hindquarter were studied using an *in vivo *constant-flow preparation.

## Results

### Growth hormone causes a transient decrease in mean arterial blood pressure and a transient increase in heart rate, Fig 1 and 2

**Figure 1:**
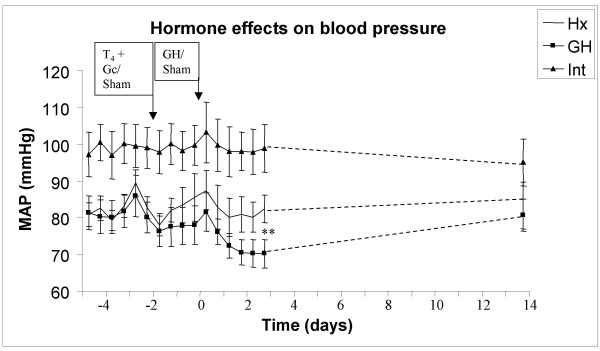
Mean arterial blood pressure (MAP) in hypophysectomized (Hx, solid line), growth hormone supplemented Hx (GH, solid squares) and in intact rats (solid triangles). The first two days (-4 to -2) represent non-supplemented basal measurements of MAP in all groups. At day -2, all Hx rats received thyroxine and glucocorticoid [T4+GC] treatment with mini-osmotic pumps, whereas the intact rats underwent sham operation. At day zero, the onset of GH treatment (mini-osmotic pumps implantation) was performed in the GH animals, whereas both intact and Hx animals underwent sham operation. * indicate p<0.05 between pre- and post-GH treatment in GH group.

**Figure 2:**
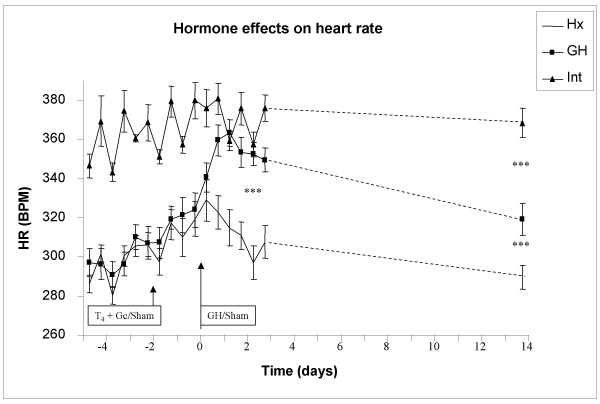
Heart rate (HR) in hypophysectomized (Hx, solid line), growth hormone supplemented Hx (GH, solid squares) and in intact rats (solid triangles). The first two days (-4 to -2) represent non-supplemented basal measurements of HR in all groups. At day -2, all Hx rats received thyroxine and glucocorticoid [T4+GC] treatment with mini-osmotic pumps, whereas the intact rats underwent sham operation. At day zero, the onset of GH treatment (mini-osmotic pumps implantation) was performed in the GH animals, whereas both intact and Hx animals underwent sham operation. * indicate p<0.05 between pre- and post-GH treatment in GH group.

Hypophysectomy *per se*, caused a 17% decrease in MAP and HR before onset of hormonal treatment (MAP; Intact: 98 ± 6, Hx: 83 ± 3, GH: 81 ± 5, p < 0.05 Intact *vs *GH or Hx, Fig 1, HR; Intact: 351 ± 7, Hx: 291 ± 4, GH: 295 ± 7, p < 0.05 Intact *vs *GH or Hx, Fig 2). Neither MAP nor HR changed when the Hx rats were treated with [T_4_+GC] compared with pre-treatment levels or with intact rats (day -2, Fig 1 and 2). Growth hormone caused an immediate drop in MAP by approx 14% (to 70 ± 4 mmHg, p < 0.05 *vs *Hx, Fig 1) with a concomitant increase in HR by approx. 16% (to 351 ± 6 beat/min, p < 0.05 *vs *Hx, Fig 2). After 14 days of GH therapy, the MAP returned to pre-treatment levels (day 14 = 81 ± 4 mmHg, Fig 1), indicating that GH had a transient effect on BP regulation. Similarly, HR returned to pre-treatment levels (pre-treatment = 319 ± 7 *vs *day 14 = 319 ± 8 beat/min, Fig 2). Mean arterial pressure was unchanged in both Hx and intact rats during the treatment period (Hx: 81 ± 3 to 83 ± 6 mmHg, Intact: 98 ± 6 to 95 ± 6 mmHg). In addition, no change in HR was detected in Hx and intact rats during the treatment period (Hx:291 ± 4 to 290 ± 6 beat/min, Intact: 351 ± 7 to 368 ± 7 beat/min). At day 14, the HR, but not MAP, was significantly changed between the groups (p < 0.05 Hx *vs *GH, p < 0.05 GH *vs *Intact and p < 0.05 Hx *vs *Intact).

### Growth hormone decreasing effect on blood pressure was NO dependent, Fig 3 and 4

#### Early L-NAME treatment

**Figure 3:**
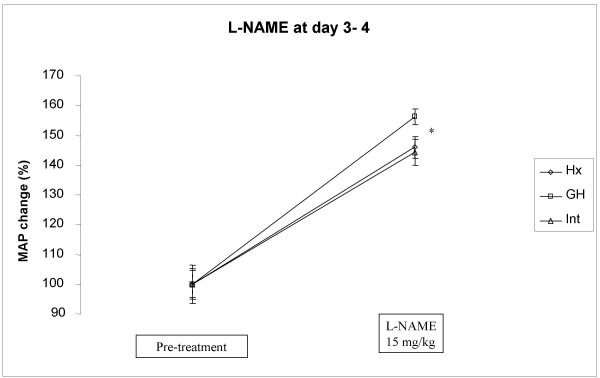
Effect of a single dos of L-NAME (15 mg/kg, s.c.) on mean arterial blood pressure (MAP) in hypophysectomized (Hx, solid line), growth hormone supplemented Hx (GH, solid squares) and in intact rats (solid triangles). The L NAME injection was given early, at day three or four (see in method section for further information) after onset of GH treatment. *indicates p<0.05 between GH and intact or Hx animals.

Both intact and Hx rats had a similar BP response to L-NAME with an increase of approx 45% (Hx from 82 ± 4 to 120 ± 7 mmHg, Intact from 99 ± 6 to 142 ± 8 mmHg, Fig 3). Growth hormone treatment caused a significantly greater BP response of approx 56% (GH from 71 ± 4 to 110 ± 6 mmHg, p < 0.05 vs. Intactand Hx, Fig 3).

#### Late L-NAME treatment

**Figure 4:**
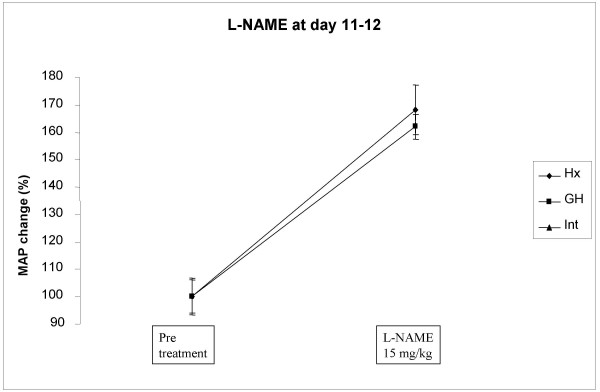
Effect of a single dos of L-NAME (15 mg/kg, s.c.) on mean arterial blood pressure (MAP) in hypophysectomized (Hx, solid line), growth hormone supplemented Hx (GH, solid squares) and in intact rats (solid triangles). The L NAME injection was given late, at day eleven to twelve (see in method section for further information) after onset of GH treatment.

There was no difference in the response of MAP to L-NAME between the groups (Intact from 91 ± 6 to 144 ± 9 mmHg, Hx from 76 ± 5 to 126 ± 5 mmHg, GH from 74 ± 4 to 120 ± 9 mmHg, Fig 4).

### Aortic eNOS expression was unaffected after 14 days of GH substitution

Hypophysectomy *per se*, had no effect on aortic eNOS protein levels (Intact: 121 ± 31 *vs *Hx:91 ± 26%). There was no significant change in aortic eNOS expression after GH treatment compared with both Hx and Intact rats (GH: 110 ± 16 %).

### Growth hormone effects on basal urine and plasma nitrate and nitrate clearance

The urine nitrate was lower, but not significantly decreased in the Hx group compared to GH and intact (Hx: 216.16 ± 72.23, Intact: 400.15 ± 94.65, GH: 641.1 ± 208.7 μM/ml/100 g BW n.s.). Both Hx and GH rats had significantly higher levels of plasma nitrate compared with intact rats (Hx: 79.4 ± 5.9, GH: 78.2 ± 5.7, Intact: 46.4 ± 2.3 μM, p < 0.05 between Hx and intact and between GH and intact). The nitrate clearance was calculated in all groups, demonstrating that GH had an increased clearance compared with Hx rats, but similar with intact rats (Hx:1.8 ± 0.6, GH: 6.2 ± 2.0, Intact: 6.2 ± 1.5 μl/min/100 g, p < 0.05 between Hx and intact and between GH and intact).

### Growth hormone caused a blunted baroreceptor activity, Fig 5

**Figure 5:**
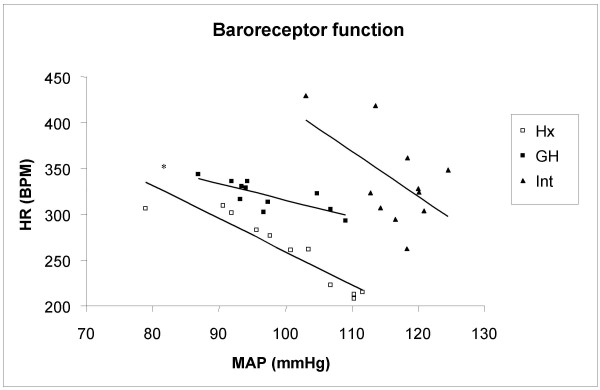
Baroreceptor activity was tested in hypophysectomized (Hx, open squares), growth hormone treated Hx rats (GH, solid squares) and in intact rats (solid triangles). Baroreceptor activity test was performed with a single dose of phenylephrine (4 mg/kg, s.c.). Mean arterial blood pressure (MAP) and heart rate (HR) were correlated to each other and the k-values were calculated in all groups. *indicate a significant difference of the slope between Hx and GH rats.

The baroreceptor activity, tested by phenylephrine, was similar in intact and Hx rats (Intact: -0.94 ± 2.29, Hx: -2.35 ± 0.69 bpm/mmHg, Fig 5). In contrast, GH resulted in a blunted baroreceptor activity (0.12 ± 0.82, p < 0.05 *vs *Hx, Fig 5). When the rats were pre-treated with L-NAME, none of the groups differed in baroreceptor activity (Intact: -2.41 ± 2.4, Hx:-2.23 ± 0.68, GH: -0.51 ± 0.40 bpm/mmHg).

### Growth hormone increases body weight

The BW of the Hx group did not change over time, indicating a complete hypophysectomy (from arrival to the onset of treatment: Intact: 295 ± 11 to 391 ± 24 g, Hx: 251 ± 19 to 225 ± 13 g, GH: 256 ± 14 to 232 ± 15 g, p < 0.05 between Intact and Hx, Intact and GH at both time points). After 14 days of treatment, the [T_4_+GC] group did not change in BW compared to pre-treated weight (Hx: 232 ± 8 to 232 ± 7 g). The Intact group increased by approx. 2% in BW during this period (391 ± 24 to 399 ± 28 g, N.S.). The GH group increased in BW by 5% during the treatment period (from 256 ± 14 to 268 ± 19 g, p < 0.05).

### Growth hormone caused a increased plasma IGF-I

Plasma IGF-I decreased in Hx rats by approx. 85% compared with Intact rats (Intact:1098 ± 46 vs Hx 164 ± 27 ng/ml, p < 0.05). There was a substantial increase in plasma IGF-I after 14 days of GH therapy by approx. 8-fold increase (Hx: 164 ± 27 vs GH: 736 ± 56 ng/ml, p < 0.05). Growth hormone treated rats displayed a significant decrease in plasma IGF-I compared with intact rats (p < 0.05).

### Growth hormone effect on heart weight and structural parameters of the resistance vessels, Table 1 and Fig 6

The relative LV weight was not changed between Hx and intact animals. Surprisingly, there was a significant loss of weight in the LV after GH therapy by approx. 2% (p < 0.05 *vs *Hx rats, Table 1). The RV weight was not changed between the groups (Table 1).

**Figure 6:**
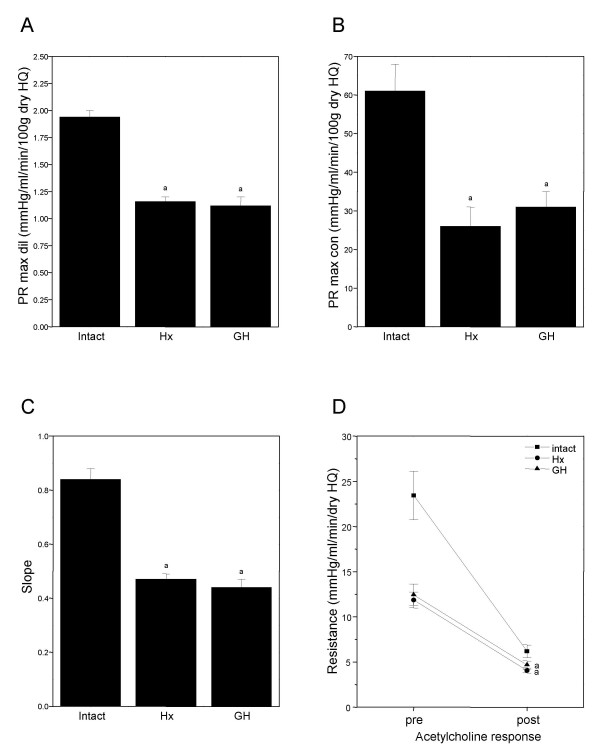
Graphs A-C showing the effect of hypophysectomy (Hx) and 14 days of growth hormone substitution to Hx rats (GH) on structural properties and acetylcholine response (**D**) in the skeletal vascular bed. (**A**) Graphs showing the perfusion resistance (PR) at maximal dilation (max dil), reflecting the average internal radius of the skeletal vasculature. (**B**) Graph showing the PR at maximal constriction (max con) obtained by barium chloride, reflecting average medial bulk of contractile tissue. (**C**) Graph showing slope, calculated by linear regression from individual pressure-flow curves during perfusion at maximal dilation. (**D**) Linear graph showing the resistance pre (during maximal noradrenaline constriction) and post (after a single dose of acetylcholine) in the skeletal vascular bed, reflecting the endothelial function. *indicate p < 0.05 between intact and GH or Hx rats.

The skeletal muscle vascular bed in Hx rats showed an increased sensitivity (left shift) to administered NA by approx. 22% compared with intact rats (Table 1), indicating an increased sensitivity of the vascular á-adrenoceptors. Treatment with GH resulted in a normalisation of the NA sensitivity (right shift) (Table 1). The perfusion resistance (PR) obtained at maximal dilation was decreased by approx. 19% in both GH and Hx group compared with intact rats (Intact: 1.94 ± 0.06, Hx: 1.16 ± 0.04, GH: 1.12 ± 0.08 mmHg/ml/min/100 g dry HQ, p < 0.05 *vs *intact and GH and Hx, Fig 6A). When vasoconstriction was induced by means of NA or with angiotensin II+phenylephrine, both Hx and GH group showed approx. 42% and 40%, respectively, decreased resistance response compared with intact rats (Table 1). Similar resistance changes were obtained in GH, Hx and intact rats after maximal constriction was induced by BaCl_2 _(Intact: 61 ± 7, Hx: 26 ± 5, GH: 31 ± 4 mmHg/ml/min/100g dry HQ, p < 0.05 Intact *vs *GH and Hx, Fig 6B). The average slope calculated from individual pressure-flow curves, also obtained during full vascular relaxation, showed similar magnitude of structural changes between Hx, GH and intact rats (Intact: 0.84 ± 0.04, Hx: 0.47 ± 0.02, GH: 0.44 ± 0.03 mmHg/ml/min/100 g dry HQ, p < 0.05 Intact *vs *GH and Hx, Fig 6C). Both the GH treated and Hx rats showed endothelial dysfunction compared with Intact rats, demonstrated by decreased dilation after a single dose of acetylcholine (Intact: 73 ± 1%, Hx: 65 ± 3%, GH: 61 ± 4%, Fig 6D).

## Discussion

The major findings in this study are; (i) Hypophysectomy *per se*, caused a decrease in MAP and HR, endothelial dysfunction and reduced vasculature mass in the hindquarter vascular bed. (ii) Supplementation with [T_4_+GC] did not change any of the studied parameters. (iii) GH substitution resulted in a transient decrease in MAP and a transient increase in HR. (iv) GH substituted rats had an increased MAP response after administration of early L-NAME compared with intact and Hx rats, indicating a transient activation of NO by GH. (v) Long-term GH treatment resulted in an impaired baroreceptor activity. (vi) 14 days of GH therapy to Hx rats neither improved the endothelial function nor restored the vascular structure. Taken together, GH substitution seems to have a short lasting effect on lowering blood pressure via activation of the NO-system. The transient increase in HR is most likely mediated by a baroreceptor activation. These data also suggest that a longer period of GH therapy is required to improve endothelial function and to restore the morphology of the vasculature. Finally, there is interplay between GH, NO-system and blood pressure regulation.

### The effect of GH on blood pressure

Growth hormone therapy to Hx rats caused an immediate and transient decrease in MAP. This reduction in MAP appears to be NO-dependent since L-NAME treatment caused a greater MAP response than it did in both Hx and intact rats. We speculate that this finding may reflect an increase in eNOS activity and/or expression, resulting in enhanced NO bioavailability. This may in turn lead to decreased total peripheral resistance [6,14], resulting in reduced MAP. Growth hormone and IGF-I has been shown to increase eNOS *in vitro *[15] as well as to increase eNOS expression [12] and NO formation [2]*in vivo*.

It has been suggested that GH treatment can be associated with an increase in extra cellular volume (ECV) [16-18]. An increased volume load may theoretically lead to an increase in BP. However, a previous published study showed that increased body sodium concentration and increased ECV after GH substitution to GHD patients were not associated with an increase in BP [18]. Taken together, this suggests that the load of ECV seems to be of minor importance for the BP regulation at least in GH treated GHD patients.

The GH activated NO-dependent decrease in MAP appears to be transient since 12 days of GH therapy, results in a similar increase in MAP after a single dose of L-NAME as it did in both intact and Hx animals. This result is supported by unchanged aortic eNOS expression in all groups and the return of MAP to pre-treatment levels in GH treated rats. The present study showed increases of urine nitrate after 12 days of GH treatment, which may suggest that there is still some GH stimulated NO production left, although this increase appears to be of minor importance for the BP regulation. The level of MAP in GH rats did not return to the level in intact rats. It is possible that the observed reduction of the resistance vessels mass in the hindquarters vascular bed could be the explanation. Folkow *et al *[13] have shown that 6 weeks of treatment with both GH and T_4 _results in normalisation of BP as well as an enhanced vascular mass in the hindquarter vascular bed. In GH over-expressed mice, increased vascular mass has also been described [19,20]. These mice displayed either hypertension [19] or normotension [20]. Thus, it seems that a longer supplementation period is required to give an effect of vascular morphology on the resistance vessels as well as to normalise BP. However, hGH treatment to Hx rats for longer periods than two to three weeks have been reported to result in antibodies against GH [21]. Guided by this information, we decided to limit the study to two weeks.

### Growth hormone effect on heart rate and the baroreceptor activity

In the present study, GH substitution results in an immediate and transient increase in HR, which is partly in accordance with other studies [2,6]. This direct effect in HR can probably be explained by an increased sympathetic activity that could originate from increased firing of baroreceptors in response to decreased MAP. Two weeks of GH therapy resulted in a normalisation of HR. This transient change in HR might be explained by deactivation of baroreceptors due to increased BP. After twelve days of GH therapy, baroreceptor activity was blunted. The blunted effect of the baroreceptors can most likely be explained in two ways; by loss of structure or by "resetting". Both GH and Hx rats showed reduced vasculature mass in the hindquarter vascular bed, but only GH treated rats exhibit impaired function of the baroreceptors, suggesting that the blunted function of the baroreceptors could not be due to loss of structure in the baroreceptors in GH rats. Therefore, our data suggest that the impaired function of the baroreceptors seems to be related to "resetting". It has been suggested that NO appears to act as a sympatholytic agent to modulate the central sympathetic outflow [22]. The impaired baroreceptor activity appears not to be NO-dependent, since the blunted effect of the baroreceptors was unchanged after L-NAME treatment.

### Growth hormone effect on adreno-receptor sensitivity and endothelial function

Growth hormone treated Hx rats showed a right shift and/or normalisation of the noradrenalin ED_50 _value in the hindquarters vascular bed, which is in accordance with other studies [11,13,19]. Long-term GH replacement in GHD patients has been shown to decrease sympathetic activity to the muscular vascular bed [23], whereas heart rate variability displays increased ratio of sympato-vagal tone, decreased vagal tone and increased sympathetic tone [24]. This might suggest that the rightward shift and/or normalisation of the noradrenalin ED_50 _value in the hindquarter vascular bed is caused by increased sympathetic tone and changed ratio between the sympato-vagal and the vagal tone.

It is well known that GHD patients exhibit endothelial dysfunction [1,25]. This phenomenon has been detected in both conduit [11] and resistance vessels in Hx rats [10]. Accordingly, in this study the resistance vessels in the hindquarter vascular bed also exhibit endothelial dysfunction in Hx rats. Neither one week [10] nor two weeks of GH therapy resulted in improved endothelial function in the resistance vessels. In contrast, both GH supplemented GHD patients [7] as well as GH treated Hx rats [11] show improved endothelial function in the conduit vessels. This suggests that longer treatment is required to enhance the endothelial function in the resistance vessels.

## Conclusion

Hypophysectomy *per se*, is associated with decreased MAP, HR and loss of vascular structure. Growth hormone substitution to Hx rats results in a transient decrease in MAP and a transient increase in HR. This transient decrease of MAP was NO dependent. Growth hormone treatment caused an impaired baroreceptor activity. Two weeks of GH therapy neither improved the endothelial function nor restored vascular parameters. To summarize, GH substitution seems to have a short lasting effect on lowering blood pressure via activation of the NO-system. These data also suggest that a longer period of GH therapy is required to improve endothelial function and to restore the morphology of the vasculature. Finally, there is an interplay between GH, blood pressure and NO-system.

## Methods

### Animals

Male Wistar rats were obtained from M&B (Ejby, Denmark). The rats underwent hypophysectomy at approximately eight weeks of age (approx. 280–300 g) at M&B one week prior arrival to our facility. The protocol conformed to guidelines on the conduct of animal experiments issued by the Swedish National Board for Laboratory Animals and was approved by the Ethics Committee for Animal Experiments at Göteborg University. The animals were housed at constant temperature (20°C) at a relative humidity of 50–60%. A 12 h dark/light cycle was maintained with lights on/off at 07.00 AM to 7.00 PM. The rats had free access to standard pellet chow and tap water throughout the study. All rats were acclimatized for one week before the onset of the experiment. Body weight (BW) was measured throughout the study once a week.

### Experimental protocol

Two different experimental protocols were used.

### Substitution therapy

In both protocol 1 and 2 (see below), all Hx rats received thyroxine (T_4_, 20 μg/kg/day) and glucocorticoid (GC, 400 μg/kg/day) ([T_4_+GC]) treatment by mini-osmotic pumps s.c. (model 2004, Alza Pharmaceuticals, Palo Alto, CA, USA). After two days of [T_4_+GC] treatment, the Hx rats were divided into two groups of which one was treated with GH (1 mg/kg/day) by a second mini-osmotic pump s.c. (2ML2) for 14 days.

### Protocol 1

Hypophysectomized rats and intact controls were equipped with telemetry transmitters for measurement of conscious unrestrained MAP and HR for 18 days (Intact n = 5, Hx n = 8, and GH n = 5). MAP and HR were measured two days before onset of treatment (Day -4 to -2) and continued throughout the start of substitution with [T_4_+GC] (Day -1 to 0) and treatment with GH (Day 1–14). In addition to these baseline measurements, two different experiments were performed; 1) the activity of the NO system was assessed by acute administration of L-NAME (early, Day 3–4) and (late, Day 11–12), 2) baroreceptor activity was assessed by acute administration of phenylephrine (Day 11–12). Two days of baseline measurements were recorded before onset of these two experiments. At the end of the 14 day protocol, rats were anesthetized, sacrificed and blood and tissues samples were taken for further analyses.

### Protocol 2

Rats in the second protocol were treated identically as in protocol 1, except that we did not implant telemetry transmitters (Intact n = 14, Hx n = 12, and GH n = 12). In this protocol, two different experiments were performed; 1) plasma and urine nitrate concentrations were measured (Day 10–12), 2) at the end of the 14 day protocol, vascular structure as well as endothelial function in the hind-quarter vascular bed was assessed using an *in vivo *constant-flow preparation.

### Experimental procedures

#### Implantation of radio-telemetric implants

During the high dose substitution therapy (see below), a radio telemetric transducer catheter (o.d. 0.76 mm, Data Science International, Inc, St. Paul, MN, USA) was implanted into the lower aorta and glued into position (3 M Vetbond™, 3 M Animal Care Products, St Paul, MN, USA) in Hx (n = 13) and control (n = 5) rats. Rats were anaesthetized using Ketalar:Rompun (39:5 mg/kg) and isoflurane (Baxter Healthcare, Chicago, MI, USA). The catheter tip was placed at least 1 cm below the renal arteries. The transmitter (TA11PA-C40) was secured to the abdominal wall and the abdomen closed with sutures. After four weeks of recovery and without hormonal supplementation, the animal in its home cage was placed on a receiver plate and the signal collected using the Dataquest LabPRO Acquisition System (Ver. 3.0, Data Sciences international, Inc, St. Paul, MN, USA). The following sampling parameters were used; sampling frequency 500 Hz, sample duration 15 sec., save period 5 min. The mean arterial signal was corrected for electronic offset, the average of one measurement outside the animal before and after implantation.

#### High dose substitution therapy to improve surgical survival

To be able to perform surgery on the vulnerable Hx rats, high-dose steroid therapy and salt (NaCl) enriched diet was given before and after surgery according to the following protocol. For five days all rats (Hx and intact rats) had free access to normal tap water and dexamethasone in tap water (orally, 1.5 mg/l). Rats also received standard pellet- and salt pellet chow (248 mmol/100 g, Lactamin, Vadstena, Sweden). The rats were also treated with glucocorticoid at day 1: 2 mg/kg × 2, day 2: 2 mg/kg × 3, day 3: (day of surgery): 2 mg/kg × 4, day 4: 2 mg/kg × 3 and day 5: 2 mg/kg × 2 (s.c.). This high dose regime was washed out during the following four weeks before onset of the experiment. During this period the rats received no hormonal treatment, but had free access to salt pellet and standard pellet chow. Using this regime 75% of Hx animals survived the surgical procedure.

### NO dependency protocol

To test if the NO system was involved in the GH-dependent BP regulation, a NOS antagonist (L-NAME 15 mg/kg, s.c.) was given during two occasions, early (day 3–4) and late (day 11–12). Saline was used as a control and given in an equal volume (0.1 ml/kg, s.c.). L-NAME and saline were given in randomized order.

### Test of baroreceptor activity

The baroreceptor activity was tested by using phenylephrine (300 μg/kg, s.c.) after pre-treatment with NaCl or L-NAME at day 11–12. Three hours after late L-NAME or saline were given; all rats received a single dose of phenylephrine to generate a slow gradual increase in MAP and a corresponding baroreceptor elicited decrease in HR within 60 seconds. L-NAME and NaCl were given in randomized order. To determine the optimal dose of phenylephrine, a dose response curve was established in separate experiments (data not shown).

### End of experiment

On day 13–14 the rats were allowed to recover and baseline measurements were performed. At day 14, the rats were anesthetized using isoflurane and decapitated. Blood samples were taken for IGF-I analysis. The heart and the aorta were quickly excised. The heart was separated into left (LV) (including septum), and right ventricles (RV) and weighed. The aorta was trimmed free of fat and adherent tissues, frozen in liquid nitrogen and stored at -80°C for further analysis.

### Measurements of IGF-I

In randomly selected plasma samples from protocol 1, the plasma IGF-I content was analyzed by a commercial RIA kit (Mediagnost, Reutlingen, Germany) in intact rats (n = 3), Hx rats (n = 5) and in GH (n = 5) treated rats [12].

### Immunoblotting

Immunoblotting and protein extraction techniques have previously been described [12]. Briefly, protein was extracted from the aorta from intact rats (n = 5), Hx rats (n = 8) and GH (n = 5) treated Hx rats. The total protein concentration was determined by a commercial protein assay (Bio-Rad, Hercules, CA, USA). 25 μg of aorta and liver of total proteins were loaded in each lane on gels (10% NuPAGE^® ^Bis-Tris gels; Novex, San Diego, CA, USA). The gels were run for 90 minutes at constant voltage (150 V). Molecular weight standards (See Blue^®^; Novex, San Diego, CA, USA) were used on each gel. The proteins were transferred to a polyvinyldifluoride (PVDF) membrane (Amersham, Buckinghamshire, UK). The membranes were incubated with a mouse monoclonal antibody against eNOS (dilution 1:1,000; Transduction Laboratories, Lexington, KY, USA). Immunoreactive protein was visualized by chemiluminescence using an alkaline phosphatase-conjugated secondary antibody (dilution 1:30,000; Sigma, St. Louis, MO, USA) and CDP-Star^® ^(Tropix, Bredford, MA, USA) as a substrate. The membranes were exposed to ECL film (Amersham, Buckinghamshire, UK) at room temperature for 1–5 minutes and the films were subsequently developed. Semi quantitative measurements of proteins from the immunoblots were made by densitometry (Fluor-S™ Multimager, Quantity One ver. 4.1.0, Bio-Rad, Hercules, CA, USA). The optical density (OD) of each band was measured. Lane containing extract of liver was used as a reference on each gel.

### Nitrate measurements

The animals were housed individually in metabolic cages. During the first day of measurement, the rats had free access to tap water and standard pellet chow. During the second and the third day in the metabolic cages, fasting was induced in the animals over night with free access to distillate water. This was in order to avoid interference of food and water on the nitrate measurement [26]. Water intake, urine volume and BW were measured. During the last day of measurements, the urine was collected for 24 hours, weighed and frozen for further nitrate analysis. At the end of the protocol, a blood sample of approximately 200 μl was drawn from the tail vein. The whole blood was centrifuged and plasma was collected and frozen for further analysis.

### Plasma and urine nitrate analyses

Plasma and urine samples were analyzed for total nitrate concentration using a gas chromatography/mass spectrometric method as previously described [27]. Briefly, after the samples had been prepared, the samples were injected into a Varian 3400 gas chromatograph equipped with a 30 m BPX-5 capillary column operated with a temperature program (60–240°C). A Varian Saturn II mass spectrometer served as detector operating in the positive ion/chemical ionization mode using methane as the reactant gas and selective monitoring of mass equivalent *m/z *124 for endogenous nitrate and mass equivalent *m/z *125 for the ^15^N-labelled internal standard. Basal urine and plasma nitrate concentrations and nitrate clearance were calculated.

### Hemodynamic analysis of resistance-vessel design

The structural characteristics of the skeletal muscle vasculature were analysed hemodynamically using a method which has been described in detail previously [13]. In brief, the isolated hind limbs of randomly treated and untreated rats were perfused in pairs via an aortic cannula. The perfusate consisted of an oxygenated 2% dextran-Tyrode solution, to which 0.5% bovine serum albumin (Sigma Chemicals, St Louis, MISS, USA) had been added. Perfusion pressure was measured via the cannulated tail artery. The hind limb vascular bed was initially maximally dilated by repeated injections of papaverine (3 mg totally), using constant flow of 10 ml/100 g hind limbs. Pressure-flow curves during maximal dilatation were constructed by altering the speed of the perfusion pump. During this constant flow condition, noradrenalin (NA) in increasing concentrations was added to the perfusate (20–64 μg). At maximal NA constriction, an injection of acetylcholine (0.01 mg, the correct dose was tested out by dose response curves, data not shown) was given. Finally, angiotensin II (200 ng), phenylephrine (4 mg) and BaCl_2 _(150 mg) were injected. Noradrenalin dose-resistance curves were constructed of hind limb vascular beds, and the ED_50 _value was calculated. After the experiment, the hindquarters were dried at 70°C for 48 h and weighed. The dry weight of the HQ was used to standardize the calculation of the hemodynamic parameters.

### Hormones, solutions and drugs

The following hormones were used; Thyroxine (T_4_, L-thyroxine; Nycomed, Oslo, Norway); Glucocorticoid (GC, cortisol phosphate; Solu-Cortef, Upjohn, Puurs, Belgium); human Growth hormone (hGH, Pharmacia, Stockholm, Sweden). Thyroxine, glucocorticoids and hGH were dissolved in 0.9% NaCl.

The composition (in mM) of the 2% dextran Tyrode solution used in the infusion experiments was Na^+ ^148.0, Cl^- ^133.4, K^+ ^4.3, Ca^2+ ^2.5, Mg^2+ ^0.8, HCO_3 _25, H_2_PO_4 _0.5, D-glucose 5.6.

The following drugs were used: acetylcholine (Sigma); Angiotensin II (Sigma); Phenylephrine (Sigma); Noradrenalin (Sigma); Nω-nitro-L-arginine methyl ester (L-NAME, Sigma); Dexamethasone (Sigma); Papaverine (Sigma).

### Statistical analysis

Values are expressed as MEAN ± SE. Statistical analyses of BW, plasma IGF-I, eNOS expression, basal urine and plasma nitrate and nitrate clearance, all HQ-data, BP and HR comparisons between the groups and percentages changes after L-NAME treatment were performed by one-way ANOVA followed by Tukey's as a post-hoc test. Hormonal effects on the BP and the HR was calculated by using paired t-test of mean of 24 h before onset of treatment compared to mean of 24 h after onset of treatment. L-NAME percentage effect on BP was calculated by comparing 10 values pre-treatment and 10 values post-treatment. Individual k-values was calculated when barorecteptor activity was studied.

## Authors' contributions

HCN: participated in the design of the study, performed some statistical analysis, acquisition of data, analysis and interpretation of data, been involved in the drafting of the manuscript

NK: carried out the nitrate measurements

KC: involved in the drafting of the manuscript

GB: participated in the design of the study, involved in the drafting of the manuscript

AW: made substantial contribution to concept and design, analysis and interpretation of data, performed statistical analysis, acquisition of data, drafting the manuscript

All authors read and approved the final manuscript

## Figures and Tables

**Table 1:** Effects of 14 days of administration of thyroxine (T4), glucocorticoids (GC) and growth hormone (GH) were studied in male hypophysectomized (Hx) rats. Intact rats were also used. The parameters that were studied were: body weight (BW), wet hindquarter (HQ weight), dry HQ, oedema, left (LV) and right (RV) ventricular and heart weight (HW) as well on structural properties in the skeletal muscle bed. The perfusion resistance (PR) was calculated as PR/flow (ml/min)/100g dry HQ weight for noradrenalin and angiotensin II (Ang II)/phenylephrine (Phe) responses. The ED-50 value demonstrates the half-maximal effect of the effective noradrenalin dose, demonstrating the adreno-receptor sensitivity. Maximal noradrenaline and Ang and Phe responses reflect the average medial bulk of contractile tissues. Values are expressed as MEAN±SE. §denotes that both protocol 1 and 2 are included. *denotes that the number in the groups are changed, intact group n=8, Hx-group n=6 and GH group n=6. adenotes p<0.05 vs. intact, bdenotes p<0.05 vs. Hx-[T4+GC].

	**Intact**	**Hx [T**_4_**+GC]**	**Hx GH+ [T**_4_**+GC]**
**N**	14	12	12
**BW (g)**	402 ± 5	222 ± 3^a^	265 ± 5^a, b^
**Wet HQ weight (g/100 g BW)**	44.4 ± 1.0	43.3 ± 3.1	41.7 ± 1.7^a^
**Dry HQ weight (g/100 g BW)**	21.6 ± 0.3	18.4 ± 0.6^a^	15.6 ± 1.7^a, b^
**Oedema (%)**	51 ± 1	57 ± 2	62 ± 2^a, b^
**LV weight (mg/100 g BW)**^§^	161.6 ± 12.6	164.4 ± 16.2	158.5 ± 14.7^b^
**RV weight (mg/100 g BW)**^§^	41.9 ± 9.9	42.1 ± 9.8	39.9 ± 9.9
**HW (mg/100 g BW)**^§^	203.3 ± 15.0	206.5 ± 20.3	186.4 ± 29.3^a, b^
**ED 50 value of noradrenalin**	0.593 ± 0.066	0.463 ± 0.05^a^	0.584 ± 0.074^b^
**PR Noradrenalin response (mmHg/ml/min/100 g dry HQ)**	23.64 ± 2.4	13.8 ± 0.8^a^	13.8 ± 1.4^a^
PR Ang II + Phe response (mmHg/ml/min/100 g dry HQ)*	33 ± 7	17 ± 2	22 ± 5

Values are expressed as MEAN ± SE. ^§^denotes that animals from both protocol 1 and 2 are included. *denotes that the number in the groups are changed, intact group n = 8, Hx-group n = 6 and GH group n = 6. ^a^denotes p < 0.05 *vs*. intact, ^b^denotes p < 0.05 *vs*. Hx- [T_4_+GC]
